# Accessory gene regulator (Agr) functionality in *Staphylococcus aureus* derived from lower respiratory tract infections

**DOI:** 10.1371/journal.pone.0175552

**Published:** 2017-04-14

**Authors:** Meissiner Gomes-Fernandes, Maisem Laabei, Natalia Pagan, Jessica Hidalgo, Sònia Molinos, Raquel Villar Hernandez, Dídac Domínguez-Villanueva, A. Toby A. Jenkins, Alicia Lacoma, Cristina Prat

**Affiliations:** 1 Microbiology Department, Hospital Universitari Germans Trias i Pujol, Institut d’ Investigació Germans Trias i Pujol, Universitat Autònoma de Barcelona, Badalona, Spain; 2 CAPES Foundation, Ministry of Education of Brazil, Brasília, Brazil; 3 Department of Biology and Biochemistry, University of Bath, Bath, United Kingdom; 4 Department of Chemistry, University of Bath, Bath, United Kingdom; 5 CIBER Enfermedades Respiratorias, CIBER, Instituto de Salud Carlos III, Badalona, Spain; Amphia Ziekenhuis, NETHERLANDS

## Abstract

**Objective:**

Characterization of *Staphylococcus aureus* clinical isolates derived from lower respiratory tract infections (LRTIs), and correlation between the functionality of the accessory gene regulator (Agr) and genotypic and phenotypic characteristics, clinical variables and clinical outcome.

**Methods:**

*S aureus* isolates derived from LRTIs and control groups (nasal carriage and bacteraemia) were genotyped using StaphyType DNA microarray. Agr activity was evaluated using the CAMP synergistic haemolysis assay and the Vesicle Lysis Test (VLT). Discordant strains were analysed by quantitative reverse-transcriptase real-time PCR (qRT-PCR).

**Results:**

Agr was functional in 79.7% and 84.5% of strains according to the CAMP and VLT assays respectively. Higher concordance with RNAIII expression measured by qRT-PCR was observed with the VLT assay (76.2%) compared with the CAMP assay (23.8%). No statistically significant differences were observed in Agr functionality between the study groups, nor the phenotypical/genotypical bacterial characteristics. No association between increased mortality/respiratory complications and Agr function was observed.

**Conclusions:**

Agr activity was high (82.2%) in isolates from LRTIs suggesting the importance of this global regulator in lower respiratory tract colonisation and infection. However, equally high Agr activity was observed in isolates derived from nasal carriage and bacteraemia, contradictory to previous observations. Agr functionality measured by the VLT assay was superior to CAMP assay.

## Introduction

*Staphylococcus aureus* is a commensal and an opportunistic pathogen, described as the causative agent in a wide range of human infections [1]. Although bloodstream and skin and soft-tissue infections represent the major burden of staphylococcal disease, lower respiratory tract infections (LRTI) by *S*. *aureus* have been increasing in the hospital setting [2, 3]. However, bacterial colonisation of the respiratory environment is known [4], making the distinction between asymptomatic colonization and infection difficult [5].

In order to establish and maintain infection, *S*. *aureus* utilizes an arsenal of virulence factors, which vary depending on its growth phase. The accessory gene regulatory (Agr) system is one of the most important and well-characterised operons in *S*. *aureus* biology, central in the control and regulation of virulence gene expression [6–9]. The operon is autocatalytic, controlled in a cell density-dependent fashion through the production and sensing of auto-inducing peptides (AIP). The Agr locus consists of two divergent transcripts, RNAII and RNAIII, driven by two promoters, P2 and P3 respectively. Activation of the P2 promoter drives the expression of the components of the quorum-sensing system (AgrBDCA). AgrB and D combine to process and secrete the AIP, which in turn acts as the ligand binding to the AgrC receptor [10]. AgrC and AgrA function as a classical two-component signal transduction system where activated AgrC phosphorylates the DNA-binding response regulator, AgrA, leading to dimerization and binding to the intergenic region between P2 and P3 promoters upregulating their expression [8]. Expression from the P3 promoter drives the synthesis of RNAIII, a highly abundant RNA effector molecule which also contains the 26 amino acid delta (δ)- haemolysin [11]. During post exponential growth, RNAIII acts reciprocally, mediating a switch from the expression of surface proteins such as protein A (SpA) to toxins and enzymes such as alpha haemolysin and proteases [11]. Airway colonization by *S*. *aureus* is a precursor for the development of LRTI, however little is known about the pathogen-associated factors that promote progression from colonization to LRTI [12]. It has been widely documented that a functional Agr is central for causing disease in animal models of infection [13–15]. However, numerous reports suggest that the role of Agr in human infection is more complex, compounded by the isolation of Agr dysfunctional strains from clinical samples [16–21]. Agr dysfunction has been associated with persistent bacteraemia, decreased susceptibility to vancomycin and thrombin- induced platelet microbicidal protein [16, 19]. Agr dysfunctional strains generally have a higher biofilm capacity and are more fit *in vitro* due to the large metabolic burden of having an active Agr system [22, 23].

Both qualitative and quantitative methods can be employed to evaluate Agr activity. The classical method of measuring Agr is via the CAMP assay, which utilises the expression of delta toxin as a surrogate for RNAIII and thus Agr function. This method relies on the synergistic haemolysis of red blood cells by delta toxin in conjunction with beta-toxin [24, 25]. Despite the straightforward handling of this assay, the interpretation of results can be subjective due to low sensitivity [17]. Recently, analytical techniques such as Whole-Cell Matrix Assisted Laser Desorption Ionization-Time-of-Flight (MALDI-TOF) mass spectrometry [26, 27] and high-performance liquid chromatography (HPLC) [28] have been successfully used to detect delta-toxin and evaluate Agr status. While both of these techniques are highly sensitive, their use for Agr evaluation has not been used frequently possibly due to high purchase costs. Detecting and quantifying Agr at the transcript level using probes (Northern blotting) or primers (quantitative reverse transcriptase-PCR (qRT-PCR)) directed against RNAIII has been used extensively [11, 29, 30]. Although both of these techniques are considerably more sensitive than CAMP, both are time consuming, laborious and expensive. A new method, the Vesicle Lysis Test (VLT), employing phospholipid vesicles containing self-quenched fluorescent dye which are specific to delta and phenol-soluble modulin toxins, acting as surrogates as RNAIII and RNAII activity has been developed [31]. Based on the current literature we decided to include the CAMP assay, as this is the conventional assay performed. In parallel, we chose to use the VLT assay as this was illustrated to be fast, highly sensitive and amenable to high-throughput. Due to the high sensitivity of qRT-PCR we chose to use this assay to evaluate Agr activity in discordant strains.

Based on the complex relationship between Agr and human infection and the lack of understanding of factors associated with *S aureus* LRTIs, the objectives of this study were two-fold. 1) To evaluate Agr function in clinical *S*. *aureus* isolates derived from LRTIs and control groups (nasal carriage and bacteraemia); and 2) to investigate correlations between Agr functionality and strain's genotypic and phenotypic characteristics, clinical variables and clinical outcome.

## Materials and methods

### Study groups and bacterial strains

*S*. *aureus* isolates included in this study were retrospectively selected from a strain collection at the Hospital Universitari Germans Trias i Pujol, a tertiary care hospital in Badalona, Spain. These isolates were obtained from intensive care unit (ICU) and non ICU patients presenting LRTIs as well as from adult nasal carriers and patients presenting bacteraemia, both representing control groups (Table 1). Ethical approval was provided by the Institutional Review Board: Comitè d' Ètica de la Investigació de l'Hospital Germans Trias i Pujol. Study was performed according to confidentiality criteria and dissociation of patients' identification data. Patient informed consent was not needed because clinical samples were not analyzed. Only clinical strains isolated during routine diagnostic tests were used, and were irreversible dissociated in order to guarantee confidentiality. All isolates were obtained from primary cultures and stored at—80°C in maintenance freeze medium (Oxoid TP, 15731) until required to minimise mutations that may affect Agr activity [17]. Strains were phenotypically characterized by conventional identification (Gram stain, selective culture media, coagulase test) and antibiotic susceptibility testing was routinely performed using the standard disk diffusion method and interpreted according to the Clinical and Laboratory Standards Institute. Strains were genotypically characterised and Agr activity was evaluated using the classical CAMP synergistic haemolysis test [17], the Vesicle Lysis Test [31] and by using real time quantitative reverse transcriptase -PCR (Real time qRT-PCR).

**Table 1:** Distribution of methicillin resistance, Agr functionality^a^ and Agr allele according to the study group considered.

Study group (n)	MRSA (%)	Agr Functionality^a^, n (%)		Allele agr, n (%)			
		Positive	Negative	I	II	III	IV
Pneumonia (22)	4 (18.2)	19 (86.4)	3 (13.6)	10 (45.5)	9 (40.9)	2 (9.1)	1 (4.5)
Tracheobronchitis (35)	8 (22.9)	27 (77.1)	8 (22.9)	15 (42.9)	14 (40.0)	5 (14.3)	1 (2.9)
Bronchial Colonization (29)	7 (24.1)	25 (86.2)	4 (13.8)	11 (37.9)	13 (44.8)	4 (13.8)	1 (3.4)
Other LRTI (9)^b^	3 (33.3)	7 (77.8)	2 (22.2)	5 (55.6)	4 (44.4)	0 (0)	0 (0)
Carriage (35)	2 (5.7)	29 (82.9)	6 (17.1)	13 (37.1)	7 (20.0)	15 (42.9)	0 (0)
Bacteraemia (18)	1 (5.6)	15 (83.3)	3 (16.7)	7 (38.9)	4 (22.2)	7 (38.9)	0 (0)
Total (148)	25 (16.8)	122 (82.4)	26 (17.6)	61 (41.2)	51 (34.5)	33 (22.3)	3 (2.0)

^a^ Agr functionality determined through CAMP, VLT and discordant strains resolved by qRT-PCR.

^b^ Other LRTI include: Pneumonitis: 1 strain; Bronchoaspiration pneumonia: 3 strains; Tracheostomy infection: 5 strains.

### Clinical definitions

Patients with LRTIs admitted to the ICU were classified according to Clinical Pulmonary Infection Score (CPIS) [32], into: bronchial colonization, tracheobronchitis or pneumonia. Other LRTI included isolates from patients presenting bronchial aspiration, tracheostomy infection and pneumonitis. Persistent respiratory isolation/bacteremia was considered as 72 hours positive cultures after patients received adjusted antibiotic treatment. Respiratory complications for patients with LRTIs were defined as respiratory failure, atelectasis, empyema, pleural effusion, pneumothorax, reinfection or shock.

### Agr CAMP assay

The conventional method used to determine the Agr functionality is via δ-haemolysin production. Agr functionality was determined by cross-streaking *S*. *aureus* isolates perpendicularly to RN4220 on sheep blood agar (SBA, Oxoid, UK). RN4220 is a *S*. *aureus* strain that produces a large zone of β-haemolysin without the interference of α- or δ-haemolysin. Plates were incubated at 37°C overnight followed by a 24 h incubation at 4°C after which they were documented. The presence of synergistic haemolysis within the β-haemolysin zone indicates the production of δ-haemolysin and, therefore, a functional Agr locus. In contrast, Agr dysfunction was defined as the complete absence of δ-haemolysin within the β-haemolysin zone. In order to establish the final result, three different observers analysed the photographs, and the presence or absence of δ-haemolysin was determined by consensus, according to Traber *et al* [17].

### Vesicle lysis test

*S*. *aureus* isolates were streaked onto tryptic Soy agar (TSA) and single colonies were transferred to 5 mL tryptic soy broth (TSB) and grown in a shaking incubator (180 rpm) for 18 h at 37°C. 1 mL of culture was harvested and centrifuged at 14000 × *g* for 10 min, and 500 μL of supernatant were recovered, stored at -20°C and lyophilized. These steps were performed in triplicate. The protocol for vesicle formulation has been described previously [31]. For the Vesicle Lysis Test (VLT), the lyophilized supernatants were resuspended with 500 μL of molecular grade water and a 50 μL aliquot was incubated with 50 μL of vesicle solution. Positive and negative controls were pure vesicles with 0.01% Triton X-100 and HEPES, respectively. The fluorescence intensity of each sample was measured for 30 min at excitation and emission wavelengths of 485–520 nm, respectively, on a FLUOROstar fluorimeter (BMG Labtech, UK). Normalized fluorescence was achieved using the equation (Ft-F0)/(Fm/F0), where Ft is the average fluorescence value at a specific time point, F0 is the minimum and Fm is the maximum fluorescence value in that particular experiment.

### Reverse transcription and quantitative RT-PCR

*S*. *aureus* isolates grown overnight in TSB were diluted 1:1000 in fresh TSB and grown at 37°C for 6 h. Cultures were normalised based on OD_600_ measurements prior to RNA isolation. Cultures were treated with two volumes of RNAprotect (Qiagen, USA) incubated for 10 min at room temperature, centrifuged at 3000 × *g* and the pellet was resuspended in Tris-EDTA (TE) buffer (Ambion, USA) with lysostaphin (Cell sciences, Canada) (250 μg/mL) and incubated for 1 h, followed by proteinase K treatment for 30 min. RNA was isolated using the RNeasy Mini Kit (Qiagen, USA) according to the manufacturer’s instructions with the addition of turbo DNase (Ambion, USA) following the purification step. RNA was quantified using RNA BR kit (Qubit, ThermoFisher Scientific, UK) and the absence of DNA was verified by PCR. Reverse transcription was performed using the QuantiTect reverse transcription system (Qiagen, USA) according to manufacturer’s instructions. Standard curves were generated for both gyrase B [*gyr*FW: 5’-CCAGGTAAATTAGCCGATTGC-3’; *gyr*RV: 5’- AAATCGCCTGCGTTCTAGAG] and RNAIII primers [*rna*IIIFW: 5’- GAAGGAGTGATTTCAATGGCACAAG-3’; *rna*IIIRV: 5’ GAAAGTAATTAATTATTCATCTTATTTTTTAGTG AATTTG-3’] using genomic DNA to determine primer efficiency. Real-time PCR was performed using the SYBR green PCR master mix (Applied Biosystems, Thermo Fisher Scientific, USA) and the Step-One Real Time PCR detection system (Applied Biosystems, Thermo Fisher Scientific, USA). Cycling conditions were 95°C for 10 min followed by 40 cycles of 95°C for 15 s and 60°C for 1 min and a dissociation step 95°C for 15 s and 60°C for 1 min. Quantities derived from standard curves were determined for 2 biological repeats and relative expression normalised to the value of the positive control (RN6390B). We determined a strain to be Agr functional with RNAIII expression levels determined to be a minimum of within 10-fold of the positive control. Isolates that had less than 10-fold level of the positive control after sampling at 6h were classified as Agr dysfunction, based on previous findings determining delayed RNAIII expression and Agr dysfunction [17, 33].

Agr functionality was categorized into positive/ negative by CAMP, VLT and/ or qRT-PCR.

### Genotypic characterization

*S*. *aureus* isolates were genotypically characterised using the StaphyType DNA microarray (Alere Technologies, Jena, Germany) [34, 35]. The Microtiter strip-mounted DNA microarrays used covered 334 target sequences (approximately 170 distinct genes and their allelic variants). PCR amplification and hybridization were performed following manufacturer’s instructions. An image of the array was recorded and analysed using a designated reader and software (Arraymate, Iconoclust, Alere Technologies) [36].

### Statistical analysis

Pearson's chi-square or Fisher test was applied when comparing categorical clinical and microbiological variables. Kappa (k) values below 0.40 indicate weak correlation, values of 0.41–0.60 indicate good agreement and values above 0.60 indicate strong agreement. Univariate and multivariate analysis as well as a logistic regression model were performed.

Associations were considered statistically significant if p value was <0.05. Data was analysed with SPSS v15 (SPSS Inc, Chicago, IL).

## Results

### Bacterial strains and study group

A total of 148 strains corresponding to 142 patients were included: 95 strains isolated from respiratory specimens (89 tracheal aspirates, 4 sputa, 1 pleural fluid and 1 protected specimen brush) from 93 patients with clinical suspicion of LRTI, 35 strains from nasal swab samples, corresponding to nasal carriers and 18 strains isolated from blood cultures.

Of the total of 148 strains included in this study, 25 (16.9%) were methicillin-resistant *S*. *aureus* (MRSA) and 123 (83.1%) were methicillin-sensitive *S*. *aureus* (MSSA) (Table 1), with 11 (8.9%) of the 142 patients designated as previous MRSA carriers. From the 111 patients presenting infection (either LRTI or bacteraemia), the origin was nosocomial (84.7%), community acquired (12.6%), and health care associated (2.7%).

High clonal complex (CC) diversity was observed among isolates included in the study. For MSSA isolates, the most frequent CCs were CC30 (22%), CC5 (17.9%) and CC45 (17.1%), whereas for MRSA isolates, CC5 (72%) was the most frequent, followed by CC22 (16%). Previous studies have highlighted that CC5 is the most frequent MRSA clonal type in Spain [37].

### Measuring Agr activity: CAMP versus VLT

The 148 isolates representing the three study groups [LTRI (n = 95), nasal carriage (n = 35) and bacteraemia (n = 18)] were assayed using both the classical CAMP assay and VLT (S1 Table), where Agr functionality was observed in 118 and 125 strains (79.7% and 84.5%) respectively. Table 1 shows the percentages of MRSA, Agr functionality and distribution of Agr alleles, in the different study groups. Concordance between both phenotypic Agr tests was 85.8% (127/148) (κ: 0.519; SE: 0.091) (Table 2). Each sample group was analysed separately: in the LRTIs group, Agr activity was functional in 77 (81.1%) and 79 (83.2%) strains using the Agr CAMP assay and VLT, respectively. Concordance between both tests was 85.2% (81/95) (κ: 0.499; SE: 0.116). Results from the nasal carriage group showed that Agr was functional in 28 (80%) and 29 (82.9%) strains using the Agr CAMP assay and VLT, respectively. Concordance between both tests was 91.4% (32/35) (κ: 0.717; SE: 0.153). Results from the bacteraemia group highlighted that Agr was functional in 13 and 17 strains (72.2% and 94.4%), respectively. Concordance between both tests was 77.7% (14/18) (κ: 0.265; SE: 0.220).

**Table 2:** Concordance between VLT test and CAMP assay results.

CAMP Assay result	VLT result		
	Positive (%)	Negative (%)	Total (%)
Positive (%)	111 (94.1)	7 (5.9)	118 (79.7)
Negative (%)	14 (46.7)	16 (53.3)	30 (20.3)
**Total (%)**	125 (84.4)	23 (15.6)	148 (100)

### Discordant cases VLT vs. CAMP assay

As shown in Table 2, a total of 21 strains presented a discordant result between both tests; LTRIs n = 14, nasal carriage n = 3, bacteraemia n = 4. Of the 21 strains with discordant results, 7 strains exhibited synergy between delta-, alpha- and beta- haemolysins on blood agar plates, and were considered as positive by the CAMP assay according to Traber *et al* [17]. However, for these strains low fluorescence intensity comparable to the negative control was observed, therefore, they were classified as negative by the VLT test. In the remaining 14 strains, the pattern of haemolysis on the SBA corresponded to an exclusive presence of beta haemolysis for 6 strains, and lack of haemolysis for 8 strains, therefore they were considered as negative by the CAMP assay. However, for these strains an increased fluorescence was measured, therefore they were classified as positive by the VLT test. To accurately determine the Agr functionality in the discordant cases, we measured the level of expression of RNAIII by real time quantitative reverse transcriptase -PCR (Real time qRT-PCR) (Table 3, S1 Fig). Of the 21 discordant strains, according to CAMP assay and RNAIII expression, a functional Agr was observed in 7 (33.3%) and 11 (52.4%) strains, respectively. Concordance between both tests was 23.8% (5/21) (κ: -0.5; SE: 0.183). According to VLT and RNAIII expression, a functional Agr was observed in 14 (66.7%) and 11 (52.4%) strains respectively, with a concordance between both tests of 76.2% (16/21) (κ: 0.516; SE: 0.18).

**Table 3:** Description of discordant cases between VLT and Agr CAMP, including haemolysin pattern, RNA III, Agr allele and clonal complex.

Study group (n) Isolate ID	VLT result	Agr CAMP result	Agr CAMP assay (haemolysin)	RNAIII expression	Agr allele	Clonal Complex
Pneumonia (2)						
S19 S116	Negative Negative	Positive Positive	Alpha and delta Alpha and delta	Negative Negative	II II	CC5 /ST5-MRSA-I CC1(ST573/772-MSSA)
Tracheobronchitis (8)						
S1 S12 S28 S44 S50 S55 S124 S132	Negative Negative Positive Positive Negative Positive Positive Positive	Positive Positive Negative Negative Positive Negative Negative Negative	Alpha and delta Alpha and delta Beta Beta Alpha and delta No hemolysin No hemolysin Beta	Negative Negative Positive Positive Negative Negative Positive Positive	I I I III I III II II	CC8-MRSA-IV [sea+], Lyon Clone/UK-EMRSA-2 CC22-MSSA CC45-MSSA CC30-MSSA CC398-MSSA [PVL+] CC30-MSSA CC5-MRSA-IV, Paediatric clone CC15-MSSA
Bronchial Colonization (3)						
S71 S145 S186	Positive Negative Positive	Negative Positive Negative	Beta Alpha and delta No hemolysin	Positive Negative Positive	II II III	CC5-MSSA CC5-MSSA CC30-MSSA
Others LRTI (1)						
S135	Positive	Negative	No hemolysin	Positive	II	CC5-MRSA-IV&VI
Carriage (3)						
S38 S39 S140	Positive Positive Negative	Negative Negative Positive	No hemolysin No hemolysin Alpha and delta	Positive Negative Positive	III III II	CC30-MSSA CC30-MSSA CC5-MSSA
Bacteraemia (4)						
S4077 S4124 S4171 S4172	Positive Positive Positive Positive	Negative Negative Negative Negative	No hemolysin No hemolysin Beta Beta	Positive Negative Negative Positive	II II III I	CC15-MSSA CC5-MSSA CC30-MSSA CC8-MSSA-SCCfus

### Correlation between Agr functionality and phenotypical and genotypical variables

Agr activity, determined through CAMP, VLT and qRT-PCR, was positive in 122 (82.4%) of the isolates (n = 148) examined. 72% of MRSA and 84.5% of MSSA isolates were Agr positive. Agr function and study groups were analysed separately. Among the 95 strains from respiratory samples, Agr was functional in 78 cases (82.1%). 22 (23.2%) out of the 95 strains were MRSA whereas 73 (76.8%) were MSSA. Regarding the 35 strains from nasal carriers, Agr was functional in 29 (82.9%) cases. 2 (5.7%) out of the 35 strains were MRSA and 33 (94.3%) were MSSA. Among the 18 strains isolated from patients with bacteraemia, Agr was functional in 15 (83.3%). One (5.6%) strain of this group was MRSA whereas 17 (94.4%) were MSSA.

We investigated potential associations between Agr functionality and the Agr allele. The most frequent group Agr allele group in the LRTIs group was AgrI (43.2%), followed by AgrII (42.1%), AgrIII (11.6%), and AgrIV (3.2%). For the strains isolated from nasal carriers, the AgrIII (42.9%) was the most frequent, followed by AgrI (37.1%) and AgrII (20%). Agr group distribution of the strains isolated from bacteraemia were both Agr I and Agr III (38.9%) and AgrII (22.2%) (Table 1). No statistically significant association was found between Agr functionality and Agr allele (Table 4).

**Table 4:** Correlation between Agr functionality determined through CAMP, VLT and qRT-PCR and genotypic characteristics, defined as presence (Pos) or absence (Neg) of specific virulence factor gene probes.

Array gene probes		LRTI (n = 95)			Carriage (n = 35)			Bacteraemia (n = 18)		
		Agr functionality			Agr functionality			Agr functionality		
		Positive, n (%)	Negative, n (%)	p-value	Positive, n (%)	Negative, n (%)	p-value	Positive, n (%)	Negative, n (%)	p-value
mecA	Pos	16 (76.2)	5(23.8)	0.519	0 (0)	2 (33.3)	0.025*	1 (6.7)	0 (0)	1
	Neg	62 (83.8)	12 (16.2)		29 (87.9)	4 (12.1)		14 (82.4)	3 (17.6)	
sarA	Pos	77 (81.9)	17 (18.1)	1	29 (82.9)	6 (17.1)	N/A	15 (83.3)	3 (16.7)	N/A
	Neg	1 (100)	0 (0)		-	-		0 (0)	0 (0)	
sak^a^	Pos	58 (81.7)	13 (18.3)	0.084	24 (82.8)	5 (17.2)	1	13 (81.3)	3 (18.8)	1
	Neg	20 (87)	3 (13)		5 (83.3)	1 (16.7)		2 (100)	0 (0)	
lukS/F-PVL	Pos	0 (0)	1 (100)	0.169	-	-	N/A	1 (100)	0 (0)	1
	Neg	78 (83)	16 (17)		29 (82.9)	6 (17.1)		14 (82.4)	3 (17.6)	
chp^a^	Pos	59 (84.3)	11 (15.7)	0.530	22 (81.5)	5 (18.5)	1	12 (80)	3 (20)	0.558
	Neg	18 (75)	6 (25)		7 (87.5)	1 (12.5)		3 (100)	0 (0)	
scn	Pos	71 (83.5)	14 (16.5)	0.253	26 (83.9)	5 (16.1)	0.546	14 (82.4)	3 (17.6)	0.833
	Neg	7 (70)	3 (30)		3 (75)	1 (25)		1 (100)	0 (0)	
tst1^b^	Pos	13 (76.5)	4 (23.5)	0.497	12 (80)	3 (20)	0.853	4 (80)	1 (20)	0.650
	Neg	65 (83.3)	13 (16.7)		16 (84.2)	3 (15.8)		11(84.6)	2 (15.4)	
Agr allele	I	33 (80.5)	8 (19.5)	0.254	12(41.4)	1 (16.7)	0.461	6 (85.7)	1 (14.3)	0.879
	II	35 (87.5))	5 (12.5)		5 (17.2)	2 (33.3)		3 (75)	1 (25)	
	III	7 (63.6)	4 (36.4)		12 (41.4)	3 (50)		6 (85.7)	1 (14.3)	
	IV	3 (100)	0 (0)		-	-		0 (0)	0 (0)	

LRTI: lower respiratory tract infection, including pneumonia, tracheobronchitis, bronchial colonization, pneumonitis, bronchoaspiration pneumonia and tracheostomy infection.

*agr*: accessory gene regulator, *sarA*: staphylococcal accessory regulator A, *mecA*: penicillin binding protein 2A, *lukS/F*-PVL: Panton Valentine leukocidin, *chp*: chemotaxis inhibiting protein, *scn*: staphylococcal complement inhibitor, *tst1*: toxic shock syndrome toxin 1, N/A: not applicable

All comparisons were done for each study group.

^a^For one strain from LRTI group an ambiguous result was obtained for the corresponding probes in the StaphyType DNA microarray.

^b^For one strain from carriers group an ambiguous result was obtained for the corresponding probe in the StaphyType DNA microarray.

* p< 0.05

When analysing together strains from the three study groups no statistical significant correlations were observed between the Agr function and methicillin resistance, clonal complex, the Agr allele, or the presence of genes encoding virulence factors. When groups analysed separately a significant association between Agr function and sensitivity to cloxacillin was observed in the nasal carriers (p = 0.025). However, this is most likely biased by the low sample number in this group (Table 4).

### Correlation between Agr functionality and clinical variables

Of the 93 patients presenting LRTI, 10 (10.8%) of them were previous MRSA carriers. Persistent isolation despite adjusted antibiotic treatment was observed in 41 (45.1%) patients. Presence of respiratory complication was documented in 18 cases (19.6%). Among the 18 patients, 5 (5.4%) fatal cases were related to respiratory infection. No statistically significant correlations were observed neither between Agr functionality and persistent isolation nor between Agr functionality and presence of respiratory complications and death. Among the 18 patients presenting bacteraemia, in 3 (16.7%) cases, positive blood culture was obtained after 72 hours. Eight (44.4%) cases developed complications: 4 patients (22.2%) presented septic metastasis and 4 patients (22.2%) presented shock. No statistically significant correlations were observed between Agr functionality and persistent blood culture after 72h, and the development of complication and/or death as shown in Table 5. No statistically significant associations were observed when a multivariate analysis was adopted to investigate the relationship between Agr function and clinical or microbiological variables.

**Table 5:** Correlation between Agr functionality and clinical variables.

Clinical variables		LRTI (n = 93)^a^			Bacteraemia (n = 18)		
		Agr functionality			Agr functionality		
		Positive, n (%)	Negative, n (%)	p-value	Positive, n (%)	Negative, n (%)	p-value
Persistent isolation after 72h^b^	No	41 (82)	9 (18)	1	13 (86.7)	2 (13.3)	0.442
	Yes	34 (82.9)	7 (17.1)		2 (66.7)	1 (33.3)	
Development of complications/mortality	No	59 (79.7)	15 (20.3)	0.509	8 (80)	2 (20)	1
	Yes	16 (88.9)	2 (11.1)		7 (87.5)	1 (12.5)	

^a^For the 93 patients in the LRTI group, persistent isolation after 72h was not considered for two cases, as these patients did not receive antibiotic treatment.

^b^ Persistent isolation after 72h despite adjusted antimicrobial treatment

## Discussion

The objective of this study was to evaluate the activity of Agr in *S*. *aureus* clinical isolates derived from lower respiratory tract, and to investigate correlations between the Agr functionality and the clinical and microbiological variables according to the different study groups. Our results illustrate a high percentage (82.1%) of LRTI isolates were Agr functional. This is in agreement with Chaffin *et al*, who observed *hld* and *psm* gene upregulation in a mouse model of pneumonia [38]. Furthermore, virulence genes regulated by Agr have an important function in escape from alveolar macrophages [39, 40] and neutrophils [41], both cells comprising the first line of defence in lung infections. Other groups have highlighted that Agr dysfunction is associated with persistent bacteraemia and that during growth in blood, apolipoproteins can interfere and inhibit quorum sensing and Agr activity [42]. Additionally, downregulating toxicity has been observed to positively influence fitness of *S*. *aureus* when grown in media supplemented with human serum [43] presumably as a trade- off to offset energetically costly toxin production in the face of a challenging environment. Recently, Painter *et al* reviewed numerous studies and concluded that strains isolated from bacteraemia displayed Agr dysfunction in 3–82% of cases [21]. This large variation may be due to differences in Agr functionality testing, isolate lineage and patient control groups. Agr dysfunction was not associated with bacteraemia strains in our study, where dysfunction was observed in 16.7% of cases.

Previous studies have questioned the sensitivity of the CAMP assay at determining Agr function [31]. Additionally, the interpretation of synergistic haemolysis can be subjective and results may vary between different laboratories, especially for clinical isolates with weak haemolytic activity [17]. To accommodate for these observations, Agr functionality was examined using both the CAMP assay and the VLT, a highly sensitive assay specific to toxins strictly regulated by Agr [31], with discordant strains measured by gold-standard qRT-PCR. Our results report a higher concordance between qRT-PCR and VLT than for CAMP assay.

There are some fundamental differences between these methods that need to be considered when evaluating Agr functionality. VLT has been shown to be highly sensitive to μM concentrations of specific Agr-regulated delta and PSMα1–4 toxins [31], a level of sensitivity which may not be achieved using CAMP assay, resulting in more isolates reported as Agr functional. The VLT is performed using harvested culture supernatant following aerobic growth in liquid broth, whereas the CAMP assay is performed under static conditions using solid agar. The difference in culture conditions may influence Agr functionality as differences in aerobic culture conditions are known to induce differential virulence gene expression [44, 45]. Additionally, the diffusion of toxins through blood agar may be less efficient at lysing cells than the interaction of toxins with vesicular lipid membranes, possibly influencing Agr functionality measurements.

To evaluate Agr function in discordant strains we investigated the expression of RNAIII, a surrogate marker of Agr function by qRT-PCR. RNA was extracted and prepared during late exponential to early stationary phase of growth (6h) where RNAIII expression is maximal [6]. Previous studies have highlighted that late transcription of RNAIII is associated with failure to translate δ- and α- haemolysin resulting in Agr dysfunction [17, 29]. Therefore, this may explain those strains that are designated Agr dysfunctional by CAMP assay but Agr functional by RNAIII measurements, as CAMP assay is dependent on synergistic haemolysis driven by toxin expression. Four strains were designated Agr functional by VLT but dysfunctional by RNAIII measurement. Recent work has shown RNAIII-independent expression of PSM toxins via AgrA binding directly to *psm* promoter [46, 47]. This may explain the above results however further work is required to fully validate this claim.

Given the importance of Agr in virulence factors expression, we investigated whether Agr function was associated with persistent respiratory isolation or complications and persistent bacteraemia. No statistical significant associations where observed in either scenario, however other factors other than Agr function may be important in persistence. *S*. *aureus* small colony variants (SCV) represent a slow growing sub-population associated with chronic, persistent and relapsing infections [48]. SCVs are phenotypically distinct, displaying reduced pigmentation, haemolysis and increased invasiveness and persistence intracellularly [48]. Recent work has observed that SCV have a rapid reversion rate when grown under laboratory culture conditions and switch to wild type phenotype upon primary culture from clinical samples [49] which may contribute to the lack of microbial factors observed in this study associated with isolates derived from persistent infections.

The importance of correctly confirming Agr functionality of a clinical isolate is highlighted by the importance of Agr for virulence in numerous animal models and associations with clinical observations. This work shows that for the majority of strains a high concordance was observed between CAMP and VLT; however, caution must be used to determine strains that are truly Agr functional/dysfunctional. Although the CAMP assay is the most routinely used to determine Agr functionality, there are pitfalls in terms of accurate measurement, ambiguous results and the impact of delayed RNAIII transcription. Higher concordance was observed between VLT and RNAIII measurements, however the high sensitivity of the VLT assay may wrongly identify Agr dysfunctional strains as functional. Therefore, we propose that a combination of phenotypic and genotypic assays must be used to accurately determine the functionality of Agr and should be used in future experiments designated to investigate associations between clinical outcome or antibiotic susceptibility and Agr function. Using a combination approach would also help to define those isolates that are completely Agr inactive (VLT negative) and those that may have low or delayed Agr activity (CAMP and RNAIII negative). The results exhibited in this study include for the first time, the analysis of Agr function in strains isolated from samples from patients with respiratory infections. However, further studies are required to determine whether the functionality of the Agr system can be a marker of pathogenicity for strains isolated from respiratory tract infection and whether this has an influence on the clinical outcome.

It is worth to mention that our cohort does not include severe necrotizing pneumonia cases, uncommon in our geographical region. On the other hand, persistence in the respiratory tract is not associated to unfavourable outcome as it is in bacteremia, but more to an adaptation to the niche [50]. Multicentric studies are already taking place in the context of the New Drugs for Bad Bugs programme, an Innovative Medicines Initiative including multiple variables (https://clinicaltrials.gov/ct2/show/NCT02413242) and will help to elucidate the real importance of bacterial molecular determinants in LRTI.

## Supporting information

S1 Table Average fluorescence values obtained by the VLT according to the study group considered. Fluorescence values obtained by the VLT for the 148 strains analysed.
(PDF)

S1 Fig Measurement of RNAIII transcription by quantitative reverse transcribed RT-PCR. RNAIII transcripts of the 21 discordant strains were evaluated following RNA extraction at 6h. Fold change in RNAIII expression has been normalised to the positive control (RN6390B*) with negative control strain (RN6911) illustrated for comparison purposes. Red line denotes the cut-off used to discriminate between Agr functional and dysfunctional strains.
(PDF)
