# Supplementary material for: Accessory gene regulator (Agr) functionality in Staphylococcus aureus derived from lower respiratory tract infections
Source: PLoS One. 2017 Apr 14;12(4):e0175552. doi: 10.1371/journal.pone.0175552 (PMC5391941; doi:10.1371/journal.pone.0175552)
Supplement: S1 Table — Fluorescence values obtained by the VLT for the 148 strains analysed. (PDF) [file pone.0175552.s001.pdf]

S1 Table. Fluorescence values obtained by the VLT for the 148 strains analysed.

LRTI (n=95)						Carrier (n=35)			Bacteraemia (n=18)		
Strain ID	Average	Error (95CI)	Strain ID	Average	Error (95CI)	Strain ID	Average	Error (95CI)	Strain ID	Average	Error (95CI)
S1	0,02456297	0,00265514	S182	0,89991233	0,00714694	S114	1,009858641	0,02247334	S4207	0,95217198	0,02832673
S10	0,79209133	0,02949091	S183	0,9931736	0,02746595	S125	0,880610104	0,0221353	S3758	0,97556236	0,02090963
S11	0,01787501	0,00337977	S184	0,89229586	0,04872532	S127	0,932508736	0,02662337	S4124	0,917556	0,03394953
S111	0,86225055	0,06735298	S185	0,97935027	0,06596011	S128	0,978575232	0,01116791	S3884	0,9804887	0,01004861
S112	0,02159244	0,00685736	S186	0,96095118	0,07533344	S139	0,964944542	0,02201264	S4143	0,94382621	0,02234311
S115	0,87375312	0,00827323	S189	0,93252387	0,04703177	S140	0,024324058	0,00473581	S4035	0,01517136	0,01455213
S116	0,26844689	0,22847196	S19	0,01025832	0,00293959	S145_33	0,077766437	0,02463041	S4145	1,02293614	0,00737643
S118	0,85050679	0,04754728	S191	0,94823515	0,05506819	S149	0,912578355	0,01832571	S3886	1,02098084	0,0331476
S12	0,03836525	0,00071547	S192	1,0388999	0,01129192	S150	0,962114602	0,01873556	S3306	1,02072815	0,0189657
S121	0,90336808	0,05958427	S193	0,80739656	0,04599483	S151	0,029470747	0,00440694	S4032	0,94757734	0,05132346
S124	0,95259713	0,06105877	S194	0,79054182	0,03990784	S152	0,976524373	0,02575922	S3750	0,99920022	0,02245283
S126	0,93004278	0,0245472	S197_1	0,93259055	0,09353579	S153	0,983250784	0,01658773	S3849	1,00869949	0,01392641
S129	0,93074142	0,03014449	S197_2	0,99108629	0,07890485	S154	0,968349246	0,0586231	S4077	1,00288266	0,02321918
S13	0,96310741	0,03815544	S2	0,86735502	0,02299982	S155	0,988629139	0,02031666	S4171	0,92708717	0,03249798
S131	0,82574439	0,0672454	S20	0,86607964	0,05607125	S158	0,906283137	0,06614062	S4172	0,95370084	0,05166634
S132	0,85500904	0,0749191	S21	0,94267302	0,01075891	S159	0,867341825	0,03273664	S4084	0,94864944	0,02000635
S133	0,82938525	0,04655242	S22	0,75181019	0,04791424	S160	0,990863578	0,03018324	S4293	0,91684208	0,047326
S135	0,87620524	0,01871512	S23	0,00406972	0,00378541	S163	1,034140629	0,03077654	S4081	0,98241456	0,04794353
S136	0,85351359	0,09985262	S28	1,03638608	0,03741302	S166_29	0,873600341	0,06483697			
S137	0,94468999	0,08805006	S29	0,50020572	0,02169529	S167_32	0,856080803	0,04901898			
S138	0,0036778	0,00044815	S3	0,89693798	0,01926997	S169	0,935557912	0,01980629			
S14	0,9162363	0,06081323	S33	0,48626612	0,00984714	S176	1,037775098	0,03426243			
S141	0,99675011	0,02388897	S34	0,0350992	0,00544157	S178	0,92085507	0,05349073			
S142	0,85933863	0,03179099	S35	0,02766245	0,0042973	S185_2	0,968519438	0,01559492			
S144	0,92387127	0,03095473	S36	0,94194734	0,00861137	S188	0,013054705	0,00270469			
S145	0,09822113	0,08805956	S37	0,86978494	0,02255845	S24	0,008883547	0,00927109			
S146	1,01319628	0,01995257	S4	0,8601248	0,03566051	S25	0,703104899	0,07459606			
S148	0,9085734	0,0235356	S41	0,93634552	0,01920548	S26	0,870779876	0,35394354			
S15	0,96357967	0,01625972	S43	0,02694003	0,00899761	S27	1,052727652	0,029553			
S157	0,80796563	0,07830949	S44	0,99002129	0,0236533	S30	1,045715842	0,04159407			
S16	0,96698732	0,05663086	S46	0,91609716	0,08014651	S31	1,045727432	0,0836321			
S162	0,91447926	0,00418077	S47	0,90356807	0,04112787	S32	0,031651658	0,01211526			
S165	0,95319833	0,03852068	S48	0,90290571	0,05024833	S38	0,873954116	0,01703038			
S166	0,89772144	0,06699516	S5	0,79743859	0,01038664	S39	1,016168969	0,00691968			
S167_1	0,88852496	0,01584848	S50	0,04086193	0,00862572	S40	0,953585401	0,0673243			
S167_2	0,91837926	0,01455271	S52	0,94633943	0,01179064						
S168	0,81527805	0,04059112	S53	0,92332028	0,08225229						
S17	0,94318592	0,02283877	S54	0,90760042	0,01049525						
S170	0,7961608	0,02128268	S55	0,80542682	0,02906871						
S177	0,02082983	0,00596207	S56	0,02356792	0,00239117						
S18	0,90871837	0,08013206	S59	0,95218021	0,04699104						
S181	0,94192278	0,0703737	S6	1,04843263	0,01188224						
S68	0,92171052	0,0139334	S60	0,83629228	0,1122626						
S69	0,92098747	0,07463326	S61	0,88018744	0,0683089						
S7	0,87770515	0,07679344	S63	0,92439831	0,06983527						
S71	0,90933004	0,0237831	S65	0,02835157	0,00351453						
S9	0,79584406	0,00921193	S66	0,82270078	0,01632382						
			S67	0,89486246	0,06221239						

Strain name, average of triplicate experiments and corresponding 95% confidence error is illustrated.
