# Supplementary material for: Accessory gene regulator (Agr) functionality in Staphylococcus aureus derived from lower respiratory tract infections
Source: PLoS One. 2017 Apr 14;12(4):e0175552. doi: 10.1371/journal.pone.0175552 (PMC5391941; doi:10.1371/journal.pone.0175552)

S1 Fig. Measurement of RNAIII transcription by quantitative reverse transcribed RT-PCR. RNAIII transcripts of the 21 discordant strains were evaluated following RNA extraction at 6h. Fold change in RNAIII expression has been normalised to the positive control (RN6390B*) with negative control strain (RN6911) illustrated for comparison purposes. Red line denotes the cut-off used to discriminate between Agr functional and dysfunctional strains.

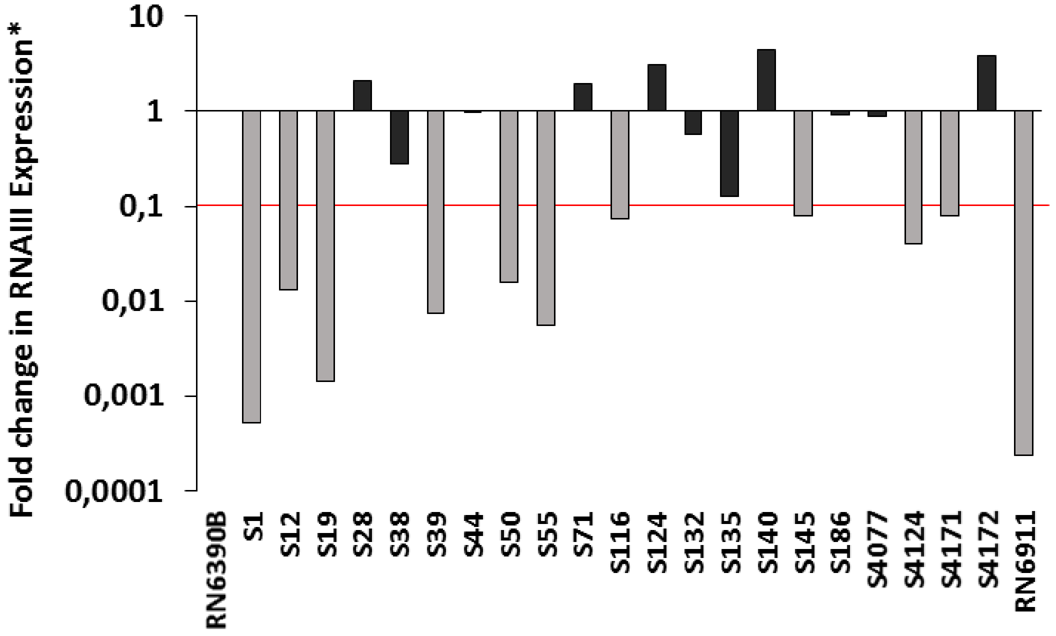

Supplement: S1 Fig — RNAIII transcripts of the 21 discordant strains were evaluated following RNA extraction at 6h. Fold change in RNAIII expression has been normalised to the positive control (RN6390B*) with negative control strain (RN6911) illustrated for comparison purposes. Red line denotes the cut-off used to discriminate between Agr functional and dysfunctional strains. (PDF) [file pone.0175552.s002.pdf]
